# Diagnostic utility of fasting versus non-fasting blood glucose: contextualising testing strategies-a narrative review

**DOI:** 10.1186/s13098-026-02213-0

**Published:** 2026-06-19

**Authors:** Richmond Owusu Ateko, Andrew Decker, Eric N. Y. Nyarko, Elikem Kumahor, Nicholas Ekow Thomford, Afua B Adjei, Justice Kumi, Samuel Mawuli Adadey, Yacoba Atiase, Henry Asare-Anane

**Affiliations:** 1 Department of Chemical Pathology, College of Health Sciences, University of Ghana Medical School, University of Ghana, Accra, Ghana; 2 Department of Psychology, School of Social Sciences, University of Ghana, Legon, Accra Ghana; 3 Division of Chemical Pathology, Department of Pathology, Faculty of Health Sciences, University of Cape Town, Cape Town, South Africa; 4 Pharmacogenomics and Genomic Medicine Group and Laboratory, School of Medical Sciences, College of Health and Allied Sciences, University of Cape Coast, Cape Coast, Ghana; 5 Department of Clinical Pathology, College of Health Sciences, Noguchi Memorial Institute for Medical Research, University of Ghana, Accra, Ghana; 6 Department of Biochemistry and Molecular Medicine, School of Medicine, University for Development Studies, Tamale, Ghana; 7 Department of Medicine and Therapeutics, University of Ghana Medical School, University of Ghana, Legon, Accra Ghana; 8 National Diabetes Management and Research Centre, Korle-Bu Teaching Hospital, Korle-Bu, Accra, Ghana

**Keywords:** Fasting plasma glucose, Non-fasting plasma glucose, Postprandial glucose, HbA1c, OGTT, Diabetes screening, Glycaemic monitoring

## Abstract

Diabetes mellitus is a chronic metabolic disorder characterised by impaired glucose homeostasis, resulting in persistent hyperglycaemia. Accurate and prompt diagnosis is essential for early intervention and prevention of long-term complications. Fasting blood glucose levels have traditionally been central to the diagnosis and monitoring of diabetes mellitus. However, growing evidence highlights the clinical relevance of non-fasting glucose measures, including postprandial glucose, random plasma glucose, and glycated haemoglobin (HbA1c) levels. Practical challenges, safety concerns, and evolving insights into glucose physiology have prompted renewed interest in flexible and context-driven approaches to glucose testing. This narrative review examines the physiological basis of fasting and non-fasting glucose regulation and critically evaluates their roles in diabetes screening, diagnosis, and monitoring. It also discusses the strengths and limitations of measuring fasting blood glucose, oral glucose tolerance, HbA1c, random plasma glucose, postprandial glucose, and continuous glucose monitoring. Special attention is given to pre-analytical and practical considerations, patient safety, and the ability of non-fasting measures to capture early metabolic dysfunction and real-world glycaemic exposure. This article reviews evidence supporting the prognostic value of postprandial hyperglycaemia and the expanding role of non-fasting monitoring tools. Non-fasting glucose testing offers substantial advantages in terms of accessibility, safety, and clinical relevance, and is well-suited for population screening and routine diabetes monitoring. Fasting glucose testing remains essential for specific diagnostic and research applications, particularly when strict metabolic standardisation is required. A context-driven framework that prioritises non-fasting approaches while reserving fasting tests for targeted indications provides a balanced and patient-centred strategy for contemporary diabetes care.

**Supplementary Information:**

The online version contains supplementary material available at https://doi.org/10.1186/s13098-026-02213-0.

## Background

Diabetes Mellitus is a metabolic disorder of carbohydrate metabolism characterised by impaired glucose homeostasis and hyperglycaemia resulting from progressive insulin resistance and relative insulin deficiency in type 2 Diabetes Mellitus (T2DM) or absolute insulin deficiency in type 1 diabetes mellitus (T1DM) [1]. Persistent elevation of plasma glucose levels is associated with complications, in particular microvascular complications [2] and may also, together with other risk factors like hypertension and dyslipidaemia, contribute to macrovascular complications [3] largely due to damage to blood vessels [4–6]. Individuals with T2DM have been reported to experience a significantly increased risk of premature mortality, with some studies estimating a reduction in life expectancy of up to 20 years [7]. The global burden of diabetes mellitus continues to rise, with the International Diabetes Federation (IDF) estimating a 45% increase in prevalence between 2024 (588.7 million people) and 2050 (852.5 million people) [8].

Glucose serves as the principal energy substrate for most cells. It is a key precursor for the synthesis of essential biomolecules, including carbohydrates, such as proteoglycans, glycogen (found in the liver and skeletal muscles), ribose and deoxyribose in nucleic acids, galactose in milk and lactose, glycoproteins, and glycolipids [9, 10]. Plasma glucose concentration is tightly regulated by complex hormonal and metabolic mechanisms, primarily involving insulin; therefore, it is a direct, clinically relevant indicator of glycaemic status. Commonly used tests include fasting plasma glucose (FPG), random plasma glucose (RPG), postprandial plasma glucose (PPG), oral glucose tolerance test (OGTT), and glycated haemoglobin (HbA1c).

These tests differ in their physiological basis and clinical applications. FPG is measured after an overnight fast of approximately 8–12 h, whereas RPG can be obtained at any time, regardless of food intake. Two-hour (2 h) postprandial glucose is conventionally measured 2 h after a meal to assess the postprandial glycaemic response. However, the timing and magnitude of peak glucose excursions vary with the glycemic index and glycemic load of the meal consumed [11, 12]. High-glycemic-load meals tend to produce earlier, sharper glucose peaks, whereas low-glycaemic-load meals lead to a more gradual, delayed rise [13, 14]. Therefore, a 2 h measurement may not capture the true glucose peak following a low-glycemic meal and measuring glucose at both 1 h and 2 h postprandially may provide a more complete picture of glycemic response, particularly in the absence of continuous glucose monitoring.

The OGTT involves first measuring fasting plasma glucose, then measuring blood glucose at 1 h and 2 h after ingestion of 75 g of oral glucose. In some clinical protocols, a 3-hour measurement is also included, particularly when assessing gestational diabetes [15]. OGTT remains the gold standard for diagnosing diabetes and the reference standard for identifying impaired glucose tolerance and gestational diabetes. HbA1c reflects average glycaemic exposure over the preceding 8–12 weeks and does not require fasting, making it a convenient tool for both diagnosis and long-term monitoring [6, 9].

Fasting plasma glucose remains the most commonly used and cost-effective test in many healthcare settings. However, its reliance on fasting introduces significant practical and clinical limitations. Adherence to fasting requirements can be challenging for patients, particularly those using insulin or oral glucose-lowering medications, and may increase the risk of hypoglycaemia. Additional barriers include long travel distances to healthcare facilities, extended waiting times in outpatient departments, and the reduced feasibility of afternoon testing due to diurnal variations in glucose levels [6]. In contrast, non-fasting glucose measurements, including RPG and HbA1c, offer greater flexibility and may facilitate opportunistic screening and earlier diagnosis, especially in resource-limited settings.

Despite the increasing use of non-fasting glucose testing in routine clinical practice, uncertainty persists about its optimal role relative to fasting measurements. Guidelines differ in their recommendations, and laboratory practices are heterogeneous. This review critically examines the evidence comparing fasting and non-fasting glucose testing, with particular attention to physiological considerations, diagnostic performance, pre-analytical factors, patient safety, and clinical applicability of the two methods. By contextualising fasting and non-fasting approaches, this review seeks to inform more flexible, patient-centred strategies for glucose testing in contemporary diabetes care.

## Methods

The primary objective of this narrative review is to evaluate the physiological and clinical differences between fasting and non-fasting plasma glucose levels and to assess their respective roles in the screening, diagnosis, and long-term monitoring of diabetes mellitus. It synthesises literature on diagnostic performance, prognostic value and practical limitations of fasting glucose tests relative to non-fasting glucose tests to contextualise current testing practices, particularly the widespread reliance on fasting glucose tests in routine clinical care. It emphasises a flexible, patient-centred framework for glucose assessment that also considers resource availability and clinical utility.

### Search strategy

Relevant literature published between 2000 and 2025 was sourced from Google Scholar, PubMed, and ScienceDirect. Search terms were developed iteratively using both Medical Subject Headings (MeSH) terms and free-text keywords, including “fasting plasma glucose”, “non-fasting plasma glucose”, “random plasma glucose”, “postprandial glucose”, “2-hour glucose”, “glycated haemoglobin”, “HbA1c”, “oral glucose tolerance test”.” OGTT”, “continuous glucose monitoring”, “CGM”, “self-monitoring blood glucose”, “SMBG”, “diabetes screening”, “diabetes diagnosis”, “glycemic monitoring”, “glycemic control”, ‘insulin resistance” and “prediabetes”. Boolean operators were used to enhance the sensitivity and specificity of the search. Additionally, articles were identified through a manual screening of the reference lists of key articles, including authoritative clinical guidelines (American Diabetes Association, International Diabetes Federation) and high-impact systematic reviews.

### Inclusion and exclusion criteria

Formal inclusion and exclusion criteria were applied flexibly, guided by the objectives of the narrative review rather than strict eligibility requirements. Priority was given to clinical practice guidelines from recognised diabetes organisations, randomised control trials, prospective and retrospective cohort studies, cross-sectional studies and systematic reviews. To ensure accurate interpretation, only studies published in English were included. Studies were excluded if they examined a specific clinical population that limited generalisability, such as organ transplant recipients, case studies or case series.

### Study selection

Titles and abstracts from the preliminary database searches were screened for relevance to the review objectives, focusing on the physiological regulation of glucose homeostasis, diagnostic performance of fasting and non-fasting glucose tests, pre-analytical and analytical considerations in glucose testing, practical implementation of glucose testing, particularly on patient safety, and technological advances in glucose monitoring. Following this initial screening, a full-text review of 225 articles was conducted, out of which 83 were selected for detailed synthesis and citation. Preference was given to recent publications (2015–2025), with rigorous methodology, and contribution to the understanding of fasting and non-fasting glucose assessment.

### Study quality assessment

The overall quality and strength of the evidence were evaluated using a hierarchical approach, assessing study design, sample size, applicability to general practice, and risk of bias. The quality of the most impactful and relevant studies included in the narrative review is summarised in Supplementary Tables 1a and 1b (ST 1a and ST 1b), and Table 1. ST 1a outlines the high-quality evidence that informed primary recommendations, classified as large randomised controlled trials, retrospective studies, prospective cohort studies with extended follow-up (> 4 years) and high-quality systematic reviews/meta-analyses. These studies had adequate statistical power, clearly defined outcomes, and appropriate control of confounding factors. ST 1b presents the moderate-quality evidence, including smaller prospective and retrospective studies (*n* = 100–2000), cross-sectional studies, and observational studies with shorter follow-up. Table 1 summarises the mechanistic and preclinical evidence provided as supporting context. These include narrative reviews (physiology, molecular biology, metabolic), guideline documents and mechanistic laboratory investigations.

**Table 1:** Mechanistic and preclinical evidence

Study	Design	Key focus	Main finding	Translational gaps and limitations
Battelino et al. 2023	Review	Standardised metrics and reporting formats for Continuous Glucose Monitoring (CGM)	Collecting at least 70% of data across 14 consecutive days can predict three-month glycemic control. A difference in Time In Range of at least 5% is considered clinically significant	There is limited evidence on the relationship between Time In Range and long-term micro- and macro-vascular complications. Studies also lack ethnic diversity
Carlson et al. 2025	Review	The efficiency of professional and personal CGM in adults with T2DM	CGM identifies more nocturnal and asymptomatic hypoglycemia than Self Monitoring Blood Glucose (SMBG). CGM is an effective educational tool that motivates behavioural changes in diabetes patients. Standardised reports from CGM can facilitate effective shhared decision making	Optimal frequency and duration for CGM check in diabetes are not yet fully established
Gracia et al., 2014	Review	Highlighting the results from a 3-year randomised clinic-based interventional study by Duran er al. (2010)	The role of SMBG in the early management and regression of T2DM	The benefits of SMBG may be lower if patients are not engaged at the onset of disease or if they experience apathy from chronic disease exposure
Heinmann 2010	Mini-Review	Drugs and other environmental factors that affect the accuracy of blood glucose meters	Acetaminophen and maltose can distort blood glucose measurements depending on the sensor’s enzyme technology. Environmental factors such as altitude and temperature, and physiological variables such as low haematocrit, can also impact the reliability of blood glucose measurements	There is limited standardisation in manufacturers’ calibration of blood glucose meters. Product labels do not meet the needs of a diverse audience
Ramljak et al. 2013	Laboratory Investigation	Assessing the interference of haematocrit on the performance of common blood glucose meters	Six out of the nineteen (32%) of the investigated meters showed stable performance across variations of haematrit. Low haematocrit (< 35%) often leads to misleadingly high glucose readings in susceptible devices.	The study used manipulated venous samples rather than capillary blood samples for which the meters are designed
Schell et al., 2013	Review	The benefits of structured and personalised SMBG	Structured SMBG significantly improves glycemic control and increases patient self-confidence and motivation	High readings may increase anxiety and self-blame in some patients
Sherwani et al., 2016	Review	HbA1c as a reliable biomarker for long-term glycemic control and cardiovascular risk	HbA1c is a strong independent predictor of coronary heart disease and stroke in both diabetic and non-diabetic subjects. It also linearly correlates with dyslipidaemia	Many low- and middle-income countries rely on unreliable or outdated HPLC devices. Variation in haemoglobin and conditions like G6PD deficiency can lead to misleading results

### Physiology of fasting versus postprandial glucose

**Fig. 1:**
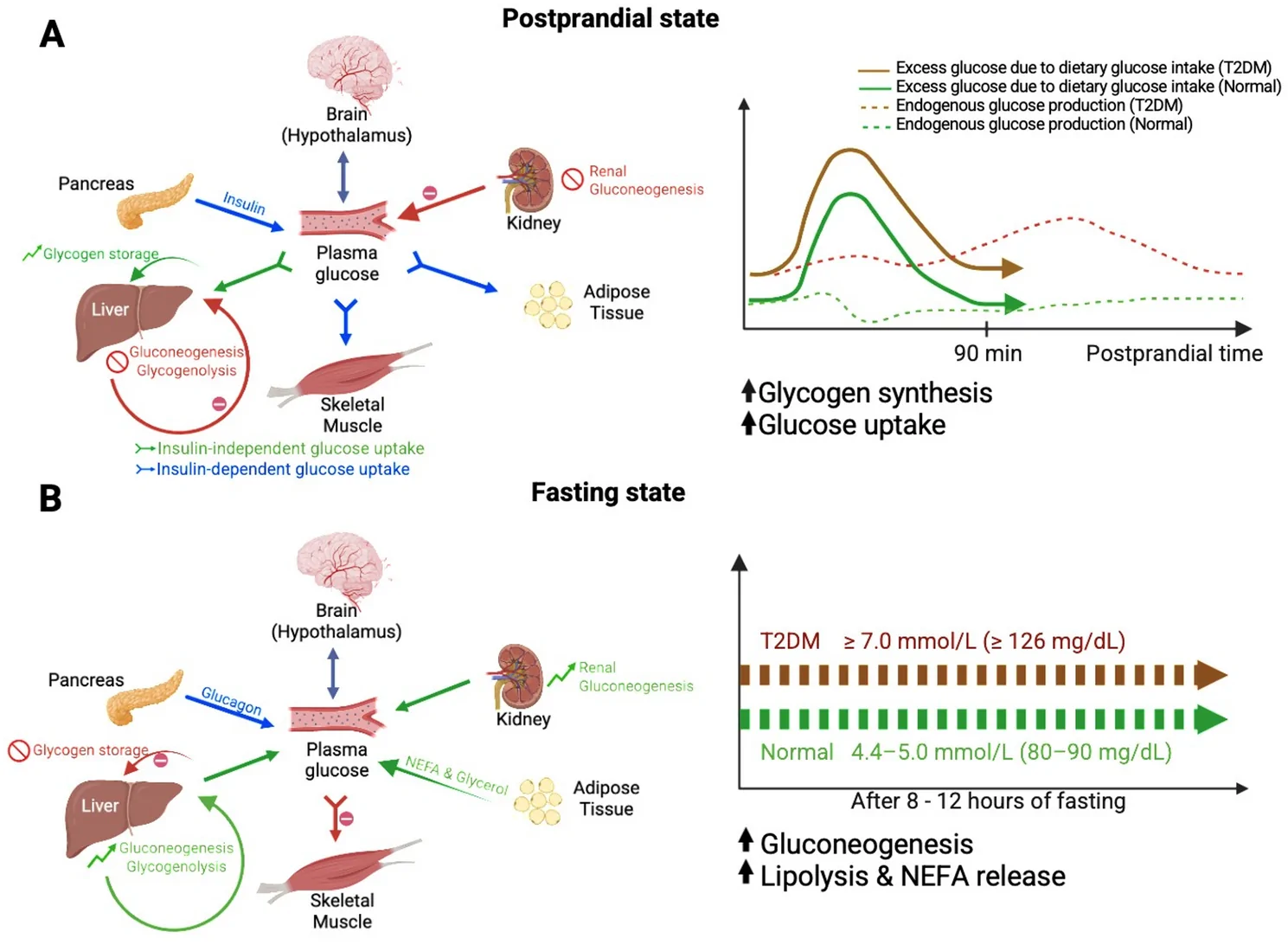
**(A)** Postprandial state showing insulin-mediated glucose uptake and glycogen storage in liver, muscle, and adipose tissue. **(B)** Fasting state showing increased gluconeogenesis, glycogenolysis, and NEFA release, supporting plasma glucose maintenance. Coordinated interactions among the pancreas, liver, skeletal muscle, kidneys, adipose tissue, and the central nervous system regulate plasma glucose homeostasis. Postprandially, insulin promotes glucose disposal and suppresses hepatic glucose production, while during fasting, endogenous glucose production sustains circulating glucose levels. Differences in glucose handling between healthy individuals and those with T2DM are highlighted

Insulin plays a central role in coordinating glucose homeostasis across multiple organ systems, whereas the liver and kidneys regulate circulating plasma glucose concentrations by controlling endogenous glucose production and peripheral glucose uptake, particularly in the skeletal muscle [16]. This integrated system also relies on adipose tissue to supply non-esterified fatty acids (NEFA) as alternative energy substrates when glucose availability declines. The physiological effects of fasting compared to postprandial glucose are shown in Fig. 1.

Following food consumption, excess glucose is taken up by the liver and stored as glycogen [17]. Hepatocytes do not require insulin for glucose uptake, unlike skeletal muscle and adipose tissue, allowing efficient postprandial glucose handling via GLUT2 transporters and glucokinase activity [18, 19]. During fasting or periods of increased metabolic demand, hepatic glycogenolysis and gluconeogenesis maintain plasma glucose availability. Gluconeogenesis is regulated by precursor availability (lactate, pyruvate, glycerol, and amino acids), pancreatic hormones, and central neural pathways involving hypothalamic insulin signalling [20–22]. Hepatic glucose production increases when insulin levels are low, permitting adipose tissue lipolysis and increasing the delivery of NEFA and glycerol to the liver. Conversely, insulin suppresses gluconeogenesis by limiting substrate availability and inhibiting the key enzymatic pathways [17]. Renal cortical cells also contribute to gluconeogenesis using lactate, glycerol, and glutamine as substrates, a process that is similarly suppressed by insulin, likely through intrarenal mechanisms rather than altered substrate delivery [23].

After approximately 8–12 h of fasting, healthy individuals typically maintain plasma glucose concentrations of 4.4–5.0 mmol/L (80–90 mg/dL) and circulating insulin levels of 7–10 µU/mL. In this state, endogenous glucose production, predominantly hepatic, closely matches peripheral glucose uptake at approximately 2 mg/kg/min, resulting in no net glucose storage [17].

In individuals with diabetes mellitus, these regulatory mechanisms become progressively impaired, leading to persistent hyperglycaemia in both the fasting and postprandial states. Disease progression in T2DM is a multistage process beginning with insulin resistance compensated by increased insulin secretion and β-cell hypertrophy, followed by a stable adaptation phase in which β-cell function can no longer fully compensate. This culminates in early decompensation, characterised by worsening fasting and postprandial hyperglycaemia and progression from pre-diabetes to overt diabetes [24].

During the early postprandial phase (first 90 min), approximately 60% of the excess glucose burden—equivalent to an additional ~ 12 g of glucose compared with non-diabetic individuals—is derived from dietary intake [25]. This reflects impaired hepatic first-pass glucose extraction in patients with diabetes. In the later postprandial phase (90 min to 6 h), sustained hyperglycaemia is primarily driven by excessive endogenous glucose production, reaching nearly twice the rate observed in healthy individuals. Similarly, fasting endogenous glucose production is approximately 30% higher in individuals with diabetes than in controls [26].

From a clinical testing perspective, postprandial hyperglycaemia indicates approximately 20 g excess glucose release over 6 h, combined with reduced glucose clearance, resulting in plasma glucose concentrations approximately 2.5-fold higher in patients with diabetes than in healthy controls. Second, the sustained elevation of blood glucose levels during the later postprandial hours reflects persistent endogenous glucose production via gluconeogenesis, which is driven by substrate availability and inadequate hormonal suppression. The severity of metabolic abnormalities, including accelerated gluconeogenesis [20], glycogen cycling [21, 22], excessive glycolysis [19], glucose clearance abnormalities [23–26] and defects in glucose oxidation [19], correlates with long-term glycaemic control indices, such as HbA1c levels. Significantly, elevated 1-hour post-load glucose concentrations are strongly associated with inflammation, cardiovascular risk, microvascular complications, and mortality [6, 27]. Moreover, blood glucose levels measured one hour after the OGTT is a strong predictor of the development of T2DM [28, 29]. Therefore, even if fasting glucose or 2-hour OGTT results are still normal, an elevated 1-hour glucose level suggests early dysregulation, typically due to early insulin resistance and/or inadequate first-phase insulin secretion.

Collectively, these findings underscore the physiological and clinical importance of assessing glycaemic control beyond the fasting state. While fasting glucose measurements primarily reflect abnormalities in hepatic glucose production, they do not capture the metabolic response to nutrient intake, including early-phase insulin secretion, glucose oxidation, and metabolic flexibility. Therefore, non-fasting glucose assessments, whether random or systematically timed, may provide a more comprehensive representation of underlying metabolic dysfunction.

This figure was created using BioRender and is available online at (https://app.biorender.com/illustrations/694c3e79669733b2b300a267).

### Diagnostic applications of fasting and non-fasting glucose testing

Approximately one-third of individuals with T2DM remain undiagnosed or are not identified until complications develop, largely because early disease may be asymptomatic or associated with non-specific symptoms [30]. This underscores the importance of effective screening strategies for detecting impaired glucose regulation before irreversible organ damage occurs. When present, clinically significant hyperglycaemia may manifest with classic symptoms such as polydipsia, polyuria, polyphagia, unexplained weight loss, fatigue, paraesthesia, pruritus, erectile dysfunction in men, and pruritus vulvae in women [31].

According to the American Diabetes Association (ADA), diabetes can be diagnosed using several biochemical criteria: fasting plasma glucose (FPG) ≥ 7.0 mmol/L (126 mg/dL), random plasma glucose (RPG) ≥ 11.1 mmol/L (200 mg/dL) in the presence of classic symptoms, OGTT ≥ 11.1 mmol/L (200 mg/dL), or glycated haemoglobin (HbA1c) ≥ 6.5% [32]. Importantly, only FPG and OGTT require fasting, whereas RPG and HbA1c can be measured in the non-fasting state, offering greater flexibility in clinical practice.

Screening for diabetes mellitus prioritises individuals at increased risk who do not yet meet the diagnostic criteria but demonstrate impaired fasting glucose (IFG) or impaired glucose tolerance (IGT). These individuals are classified as having pre-diabetes, an intermediate metabolic state associated with a substantially increased risk of progression to T2DM. Longitudinal studies indicate that approximately 5–10% of individuals with pre-diabetes progress to diabetes annually [33]. While fasting tests such as FPG and OGTT have traditionally been used to identify IFG and IGT, non-fasting measures—including postprandial glucose and HbA1c—are increasingly recognised as valuable tools for identifying early dysglycaemia.

From a diagnostic perspective, fasting and non-fasting glucose tests provide complementary information. Fasting measurements primarily reflect the abnormalities in basal hepatic glucose production. In contrast, non-fasting and postprandial glucose measurements detect defects in insulin secretion, peripheral glucose uptake, and metabolic responses to nutrient intake. Each diagnostic test for fasting and non-fasting plasma glucose, along with its application, is presented in Table 2.

**Table 2:** Diabetes diagnostic tests

Test Name	State	Diabetes threshold	Pre-diabetes threshold	Application & clinical use
**Fasting Plasma Glucose (FPG)**	Fasting (8 + hours)	≥ 126 mg/dL (7.0 mmol/L)	100–125 mg/dL (5.6–6.9 mmol/L)	Standard screening tool; measures basal (overnight) glucose control.
**HbA1c**	Non-fasting	≥ 6.5% (48 mmol/mol)	5.7%–6.4% (39–47 mmol/mol)	Measures average blood sugar over 2–3 months; highly convenient as it requires no preparation.
**Oral Glucose Tolerance Test (OGTT)**	Fasting, then sugar drink	≥ 200 mg/dL (11.1 mmol/L)	140–199 mg/dL (7.8–11.0 mmol/L)	Evaluates the body’s peak response to sugar; critical for diagnosing gestational diabetes.
**Random Plasma Glucose**	Non-fasting	≥ 200 mg/dL (11.1 mmol/L)	*N/A (not used for prediabetes)*	Used in patients with classic symptoms of hyperglycemia (e.g., extreme thirst, frequent urination).

HbA1c integrates both fasting and postprandial glycaemic exposure over time and does not require fasting, making it particularly useful for population screening and routine clinical assessments. Therefore, understanding the appropriate application of each test is essential for accurate diagnosis and timely intervention. The continuum from normoglycaemia to diabetes, based on commonly used diagnostic thresholds, is presented in Table 3.

**Table 3:** Plasma glucose and HbA1c thresholds for diabetes diagnosis

Glycaemic category	Fasting blood glucose (mmol/L)	Postprandial blood glucose (mmol/L)	HbA1c (%)
Normal	< 5.6	< 7.8	< 5.7
Pre-diabetes	5.6–6.9	7.8–11.0	5.7–6.4
Diabetes	≥ 7.0	≥ 11.1	≥ 6.5

### Pre-analytical and analytical considerations

The accuracy of blood glucose measurement depends on analytical performance, proper test execution and awareness of pre-analytical factors that may compromise the results. These considerations are particularly relevant for self-monitoring of blood glucose (SMBG) and capillary point-of-care testing, where user-related errors account for up to 90% of the observed measurement discrepancies [34]. Self-monitoring blood glucose involves intermittent capillary glucose measurements performed by patients throughout the day, most of which are obtained in the non-fasted state. Contamination of test strips, especially by glucose-containing substances, can substantially elevate readings, as shown when patients fail to wash their hands before testing [35]. Such errors are strongly linked to inadequate patient education, with studies demonstrating that up to 69% of individuals who initially perform tests incorrectly achieve acceptable results after targeted re-education [36].

The timing of glucose measurement and the patient’s physiological state are additional important pre-analytical variables [37, 38]. Prolonged fasting periods place insulin-treated individuals at an increased risk of hypoglycaemia, particularly when fasting requirements are extended due to delays in sample collection. In capillary testing, peripheral perfusion also influences measured glucose concentrations, as reduced blood flow to peripheral tissues can bias point-of-care readings [39, 40]. Previous studies [41] have also reported that haematocrit deviations below 35% can impact blood glucose test results. Most glucometers are calibrated to assume a normal haematocrit range of between 35% and 50%; therefore, in cases of reduced red cell volume, such as anaemia, the relative plasma volume increases. This can lead to an overestimation of the glucose concentration because glucose is dissolved in the plasma. Lipaemic samples arising from elevated triglycerides may also systematically bias blood glucose results by interfering with glucometers’ measurement systems [42]. Medication use may complicate the interpretation of glucose measurements. Acetaminophen has been shown to cause falsely elevated readings in some electrochemical glucose monitoring systems [37]. Icodextrin, a glucose polymer used in peritoneal dialysis solutions, can interfere with both glucose oxidase- and glucose dehydrogenase-based technologies, resulting in spurious hyperglycaemia [38]. Awareness of such interferences is essential when interpreting both fasting and non-fasting glucose results.

Non-fasting glucose testing offers practical advantages by eliminating the risks of prolonged fasting while maintaining clinical utility, provided that the results are interpreted in relation to recent food intake. The use of standardised descriptors, such as a clearly defined 2-hour postprandial measurement, improves comparability across testing occasions and clinical settings [43, 44]. Ultimately, optimal glucose testing strategies should balance analytical precision with practical feasibility and patient safety, reinforcing the rationale for a more flexible and context-driven approach to fasting and non-fasting glucose assessment.

### Long-term monitoring of glycemic control in diabetes

While screening and diagnostic testing aim to identify individuals with diabetes, effective long-term management of this chronic condition requires ongoing assessment of glycaemic control. Glycated haemoglobin has emerged as the standard of care for monitoring diabetes and guiding therapeutic decisions. Its clinical value lies in its ability to reflect average glycaemic exposure over time and its well-established association with microvascular complications [45]. Population-based studies have further demonstrated strong associations between HbA1c levels and cardiovascular disease risk and all-cause mortality [46]. A 1% increase in HbA1c has been associated with an approximately 30% increase in all-cause mortality among individuals with diabetes, whereas modest improvements of 0.2% have been linked to meaningful reductions in mortality risk [47].

Importantly, HbA1c measurement does not require fasting and can be performed at any time of the day, avoiding diurnal variability and logistical constraints associated with fasting plasma glucose testing [48]. HbA1c results are routinely used to guide pharmacological therapy and lifestyle interventions, including dietary modifications and physical activity. Ongoing standardisation of HbA1c through the National Glycohaemoglobin Standardisation Program (NGSP) has improved analytical accuracy and comparability across laboratories [49]. Current recommendations suggest measuring HbA1c at least twice yearly in patients with stable glycaemic control and more frequently in those changing therapy [50]. Nevertheless, the utility of HbA1c is limited in certain situations, such as conditions affecting red blood cell turnover, haemoglobin variants, and the influence of some medications, with ongoing debate regarding optimal clinical thresholds [30].

These limitations have contributed to an increased reliance on self-monitoring of blood glucose (SMBG) and continuous glucose monitoring (CGM). Self-monitoring blood glucose is performed by patients throughout the day, most of which are obtained in the non-fasted state. Unlike fasting plasma glucose, SMBG provides glucose values within the context of meals, physical activity, and daily routines, offering insights into short-term glycaemic variability [51]. However, SMBG requires frequent finger-prick testing, which may cause discomfort and reduce adherence, and the interpretation of results depends heavily on patient education [52, 53]. Insufficient measurement frequency may also limit the usefulness of the CGM in guiding basal insulin adjustments [54].

Continuous glucose monitoring represents a recent advance in glucose monitoring and involves continuous measurement of interstitial glucose via a wearable sensor. This approach provides a comprehensive profile of glycaemic patterns throughout the day and night, capturing trends and fluctuations that may be missed by intermittent testing [55]. CGM systems generate clinically meaningful metrics, such as time-in-range, time spent above or below target ranges, glucose variability, and estimated average glucose, which correlate closely with HbA1c [56]. By capturing responses to meals, physical activity, psychosocial stressors, and medications, CGM facilitates precise therapeutic adjustments and personalised lifestyle interventions [54]. However, high device costs remain a significant barrier to widespread adoption, particularly in low-resource settings [57].

In practice, combining multiple monitoring modalities may be ideal. HbA1c provides an integrated assessment of long-term glycaemic control and remains the primary outcome measure in clinical trials, whereas SMBG and CGM offer granular, predominantly non-fasting data that support day-to-day clinical decision-making. Although fasting plasma glucose remains useful for assessing basal glycaemia, particularly in insulin-treated patients, it is insufficient as a sole tool for ongoing diabetes monitoring [54].

### Advantages and limitations of fasting and non-fasting plasma glucose testing

The key advantages and limitations of fasting and non-fasting glucose testing across clinical and health system domains are summarised in Table 4.

A key advantage of FPG testing is that by requiring patients to abstain from food for a defined period (typically 8–12 h), it provides a standardised metabolic context for assessment [10, 17]. During this interval, glucose homeostasis is relatively stable, allowing the results to be compared across individuals, time points within the same individual, and populations. This reproducibility has enabled the establishment of diagnostic thresholds based on disease risk, supported by studies linking fasting hyperglycaemia to microvascular and macrovascular complications [58, 59]. Fasting plasma glucose also provides specific insights into basal hepatic glucose production and the adequacy of insulin action. Therefore, elevated fasting glucose reflects relatively advanced metabolic dysfunction, although it may be less sensitive in detecting early dysglycaemia [6, 28, 60].

However, these advantages are accompanied by significant limitations. Fasting requirements impose a logistical burden on patients, particularly those with work, caregiving, or transportation constraints, and may reduce participation in tests [30, 61, 62]. Patient safety is another concern; individuals treated with insulin or insulin secretagogues face a genuine, albeit uncommon, risk of hypoglycaemia during prolonged fasting [54, 63]. Additionally, the discomfort and inconvenience of fasting may cause patients to defer testing [64]. Furthermore, although FPG levels demonstrate reasonable reproducibility, biological variability remains significant. A substantial proportion of individuals with elevated fasting glucose levels on initial testing may have normal values on repeat measurements, highlighting the intra-individual variability that cannot be ignored [59, 65].

Fasting is mandatory for the OGTT. This test depends on establishing an actual baseline fasting state before ingesting a standardised 75 g glucose load, with plasma glucose measured one or two hours thereafter [61, 66]. The OGTT remains the gold standard for diagnosing gestational diabetes mellitus (GDM) and is typically performed between 24 and 28 weeks of gestation. GDM affects approximately 3–9% of pregnancies globally, depending on ethnicity, maternal age, and obesity prevalence [67], and early identification is essential to reduce the maternal and foetal risks [63]. In non-pregnant individuals, OGTT is valuable for confirming impaired glucose tolerance when fasting glucose and HbA1c levels are inconclusive, particularly in those with a strong family history of diabetes, prior GDM, or polycystic ovary syndrome (PCOS) [34, 68, 69]. In research settings, the standardised conditions of the OGTT make it an important outcome measure for cross-population comparisons and evaluation of pharmacological interventions [70, 71]. However, OGTT is time-consuming, sensitive to recent diet, physical activity, illness, and medication use, and may be poorly tolerated due to gastrointestinal side effects [28, 34, 62]. OGTT is also known to demonstrate some variability. Approximately 20% of OGTT tests yield nondiagnostic or inconsistent results [72]. Repeat testing is recommended, unless the initial findings are unmistakably abnormal. To minimise this variability, patients should avoid caffeine and medications known to interfere with glucose metabolism, especially the evening before testing. Similarly, patients should avoid excessive physical activity before testing because exercise enhances skeletal muscle glucose uptake and reduces insulin resistance, potentially blunting post-load glycemic excursions [73]. Additionally, OGTT is best conducted in the morning, as glucose tolerance is known to have diurnal variations in insulin sensitivity [74]. Similar preanalytical considerations are relevant for standardised 2 h postprandial glucose measurements, since diet, physical activity, medications and circadian variation can all influence postprandial glycaemic excursions and therefore distort test results [72]. When fasting is required, patient safety should remain paramount, including appropriate counselling on the adjustment of insulin or sulfonylurea therapy [53, 63]. Therefore, fasting protocols should be applied selectively, based on clear clinical or analytical justification, rather than as routine practice.

Many of these limitations are mitigated by non-fasting glucose testing, which offers greater convenience, flexibility, and accessibility by allowing testing at any time of the day. Removing the requirement to fast reduces the risk of hypoglycaemia and improves patient participation. Importantly, non-fasting glucose measurements capture metabolic function under real-world conditions [75], which is clinically relevant, given that postprandial dysglycaemia often precedes fasting hyperglycaemia in the pathogenesis of T2DM. Therefore, non-fasting testing may be beneficial for the early detection of metabolic dysfunction.

HbA1c provides an integrated measure of average glycaemia over approximately 8–12 weeks by quantifying non-enzymatic glycation of haemoglobin [76]. In addition to not requiring fasting, HbA1c exhibits lower intra-individual variability than single glucose measurements and is relatively unaffected by acute stress or illness [77, 78]. However, conditions affecting red blood cell turnover, such as haemolytic anaemia, recent transfusion, chronic kidney disease, and pregnancy, may lead to misleading results [6, 79–81]. HbA1c is also less sensitive than glucose-based tests for detecting early diabetes and is associated with higher analytical costs [30].

Random plasma glucose (RPG) has proven valuable for opportunistic screening in primary care and community settings, where fasting samples are impractical [82]. However, RPG values must be interpreted cautiously, as glucose concentrations vary substantially after recent food intake. Therefore, diagnostic use requires the presence of classic hyperglycaemic symptoms or confirmatory testing. This inherent variability has driven interest in standardised postprandial testing, typically performed 2 h after food intake. However, as noted earlier, meal composition and overall glycemic load significantly influence glucose peaks. Lower-glycemic-load meals potentially reduce the sensitivity of isolated 2-h postprandial measurements [83]. A meta-analysis by Toh and colleagues (2020) found that lowering meal glycemic index and glycemic load significantly attenuated postprandial glucose responses at 60, 90 and 120 min [84]. Although 2 h postprandial glucose measurement is commonly used in clinical practice to assess therapeutic response, with target values generally **<** 7.8 mmol/L in individuals with established diabetes [85], in the absence of continuous glucose measurement, postprandial glucose measurements at both 1 and 2 h may provide a more comprehensive assessment of postprandial dysglycemia.

Taken together, these considerations underscore that the optimal approach to glucose testing is context dependent. For population screening, opportunistic case-finding, and routine monitoring of glycaemic control, non-fasting testing offers clear, practical, and safety advantages. Conversely, the standardisation afforded by fasting glucose measurements remains valuable for diagnostic confirmation, particularly when results are near the diagnostic thresholds or when assessing specific metabolic abnormalities, such as impaired fasting glucose.

**Table 4:** Advantages and limitations of fasting and non-fasting glucose testing

	Fasting glucose testing (e.g. FPG, OGTT)	Non-fasting glucose testing (e.g. RPG, postprandial glucose, HbA1c, CGM)
Advantages	• Reflects basal hepatic glucose production and fasting insulin action • Its standardisation allows for easy comparison across studies • Useful for confirmatory diagnosis and assessment of impaired fasting glucose • It is essential for OGTT	• Captures postprandial glycaemic responses, daily variability, and real-world metabolic function • Higher sensitivity, particularly for postprandial hyperglycaemia • Reduced hypoglycaemia risk due to the absence of prolonged fasting • Central role via HbA1c, SMBG, and CGM • Flexible workflow; facilitates large-scale screening and follow-up • High convenience; can be performed at any time of day • Suitable for opportunistic screening, early detection of dysglycaemia, and diagnosis when criteria are met • Highly suitable due to flexibility, safety, and accessibility • Higher sensitivity for postprandial hyperglycaemia
Limitations	• Lower sensitivity; abnormalities often appear later in disease progression • Lower convenience; requires fasting and early scheduling • Risk of hypoglycaemia in insulin- or sulfonylurea-treated patients • Affected by fasting duration, diurnal variation, and medication timing • Limited role; mainly reflects basal glycaemia • Requires scheduling and patient preparation; may reduce throughput • Logistically challenging due to fasting requirements	• Greater physiological variability; interpretation requires contextual information • Affected by meal timing, composition, physical activity, and stress

### Clinical implications and future perspectives

**Fig. 2:**
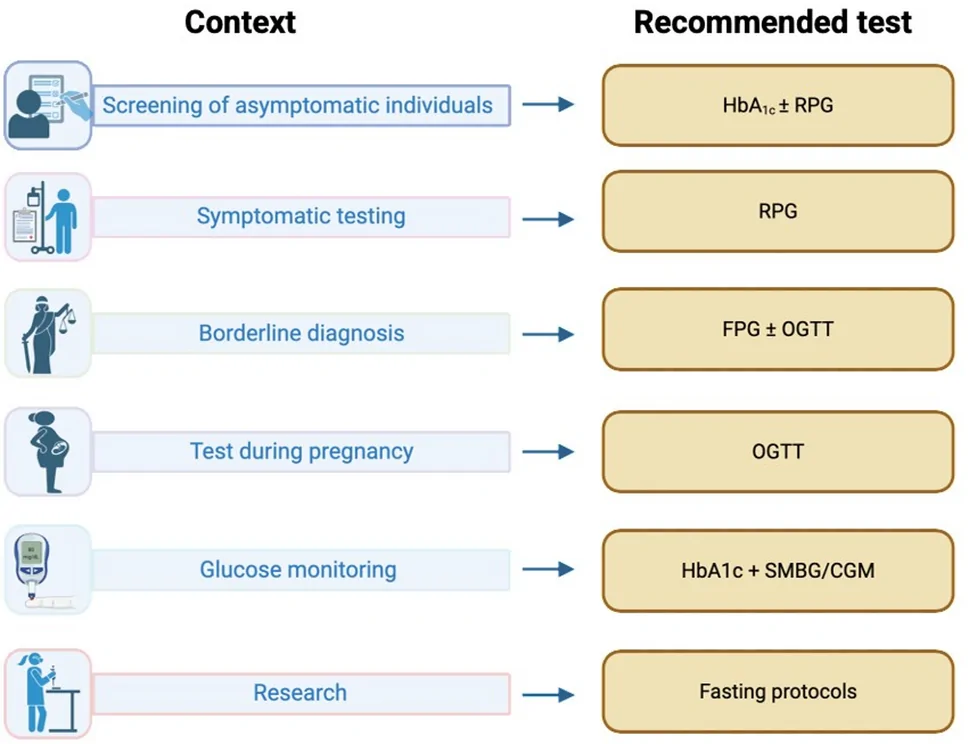
Context-driven framework for glucose testing

This overview presents information supporting a growing transition in clinical practice toward a more adaptable, context-specific methodology for blood glucose testing. The physiological intricacy of glucose metabolism necessitates a diverse approach to dietary health, which involves an awareness of and potential use of fasting regimens, rather than their elimination. The body’s complex, redundant energy management systems (metabolic flexibility) were shaped by evolutionary cycles of feast and famine, and fasting activates these deep-seated mechanisms in ways that continuous feeding does not. In contrast, non-fasting measures, including random plasma glucose and HbA1c, can be prioritised for population screening and opportunistic case-finding among asymptomatic individuals (Fig. 2), thereby reducing barriers to participation and improving early detection. Expanding access to non-fasting testing has the potential to enhance diabetes case-finding in underserved populations and facilitate earlier intervention when lifestyle and pharmacological measures are most effective.

For longitudinal monitoring, HbA1c, when combined with continuous glucose monitoring, provides a comprehensive assessment of glycaemic control without the need for fasting samples [6, 17]. This approach reflects real-world glycaemic patterns and aligns with patient-centred care. However, HbA1c has important limitations in clinical scenarios involving patients with haemoglobinopathies, such as sickle cell disease, thalassaemia, and hereditary spherocytosis, due to altered red blood cell turnover and lifespan [86–88]. These diagnoses contribute to HbA1c result distortions. Moreover, conditions that impact red blood cell survival, such as recent blood transfusions and chronic kidney disease, can also compromise the reliability of the HbA1c test [89, 90]. Scenarios such as these, and alternative biomarkers such as glycated albumin or fructosamine, hold significant value because their results are independent of red blood cell concentration [91, 92]. However, for implementation to be successful, medical personnel must be sufficiently trained to assess non-fasting glucose results in relation to dietary intake, nutritional composition, physical activity, and clinical situation. The adoption of standardised reporting descriptors, such as clearly defined postprandial time points, would further enhance interpretability and consistency across clinical settings [93].

In the future, the development of validated thresholds for random and standardised postprandial blood glucose measurements that predict diabetes risk and clinical outcomes comparable to fasting glucose criteria would promote the broader implementation of no-fasting glucose testing. Achieving this goal will require coordinated efforts from cross-regional, multidisciplinary expert panels, supported by high-quality systematic reviews and pooled analyses that account for population heterogeneity. Particular attention should be paid to evidence from low- and middle-income settings, where the practical advantages of non-fasting testing may be more significant. Regular re-evaluation of diagnostic thresholds as new evidence emerges will be essential to maintain scientific rigour, clinical relevance, and equity in diabetes care [6].

This figure was created using BioRender and is available online at (https://app.biorender.com/illustrations/694c3e79669733b2b300a267).

## Conclusion

Overall, the evidence supports a context-sensitive approach to glucose testing, in which fasting and non-fasting measures – including RPG, postprandial glucose measurements, and HbA1c- each provide clinically valuable information for the early detection, diagnosis, and monitoring of dysglycaemia.

### Limitations

Several potential limitations emerge from both the subject matter examined and the narrative review approach. By design, the narrative review prioritised landmark studies and recent publications, contributing to the risk of selection bias. Efforts to limit this risk included conducting a comprehensive search of relevant literature across multiple databases, incorporating both supportive and contradictory evidence, and drawing on systematic reviews to identify additional key studies. Nevertheless, the possibility remains that certain viewpoints may have been underrepresented.

Another limitation is the heavy reliance on preclinical evidence, which limits the strength of inferences that can be made regarding clinical decision-making, the diagnostic performance, and the prognostic value of glucose testing strategies. The predominance of preclinical data in the included literature precluded conducting a quantitative synthesis or meta-analysis. However, this approach enabled consolidation of evidence across mechanistic experiments, small clinical studies and large epidemiological cohorts- sources that would not be methodologically appropriate to combine quantitatively.

## Supplementary Information

Below is the link to the electronic supplementary material.


Supplementary Material 1.


## Data Availability

Not applicable. No new datasets were generated or analysed in the preparation of this review.

## Abbreviations

ADA: American Diabetes Association
CGM: Continuous Glucose Monitoring
FBG: Fasting Blood Glucose
GDM: Gestational Diabetes Mellitus
HbA1c: Glycated Haemoglobin
IDF: International Diabetes Federation
IFG: Impaired Fasting Glucose
IGT: Impaired Glucose Tolerance
NEFA: Non-Esterified Fatty Acids
NGSP: National Glycohaemoglobin Standardisation Program
OGTT: Oral Glucose Tolerance Test
PCOS: Polycystic Ovary Syndrome
PPG: Postprandial Plasma Glucose
RPG: Random Plasma Glucose
SMBG: Self-Monitoring Blood Glucose
SP: Supplementary Table
T1DM: Type 1 Diabetes Mellitus
T2DM: Type 2 Diabetes Mellitus
