# Supplementary material for: Diagnostic utility of fasting versus non-fasting blood glucose: contextualising testing strategies-a narrative review
Source: Diabetol Metab Syndr. 2026 Jun 19;18:190. doi: 10.1186/s13098-026-02213-0 (PMC13531804; doi:10.1186/s13098-026-02213-0)
Supplement: Supplementary file 1 [file 13098_2026_2213_MOESM1_ESM.docx]

Supplementaer Table 1a (SP 1a): High-quality Evidence

| **Study** | **Design** | **Sample Size** | **Key Outcome** | **Main Finding** | **Limitations** |
| --- | --- | --- | --- | --- | --- |
| Ahuja et al., 2021 | Meta-analysis | 35,551 | The optimal cut-off 1-h post-load glucose for predicting T2DM | 1-h postprandial glucose of ≥ 11.6 mmol/L during OGTT showed good sensitivity (0.92) and specificity (0.91) | The total number of studies included was relatively small and the funnel plot showed asymmetry, indicating sampling bias. Bias may also arise from the use of aggregate measures. |
| Cavelot et al., | Prospective cohort | 505 | Cardiovascular events and all-cause mortality | 2-h Postprandial glucose and HbA1c predict cardiovascular events and all-cause mortality in T2DM | Causality can not be proven due to the epidemiological nature of the study |
| Khaw et al., 2001 | Prospective population study | 4,662 | Mortality, all-cause, cardiovascular disease and ischemic heart disease | 1% increase in HbA1c is associated with a 28% increased risk of death | Analysis was restricted to male participants due to insufficient events in women for robust analysis |
| Kim et al., 2023 | Retrospective cohort | 2,801,153 | Sudden Cardiac Arrest | Long-term increases in fasting plasma glucose are significantly associated with an increased risk of sudden cardiac arrest in people with diabetes | The cohort was predominantly of East Asian descent. Reliance on claims-based data has the inherent risk of coding errors |
| Pareek et al., 2018 | Prospective population Cohort | 4,867 | Predicting T2D2, vascular complications and mortality | 1-hour post-load glucose is a stronger predictor of T2DM, complications and mortality than 2-h | Gender bias in the sample with male participants outnumbering female participants |
| Selvin et al., 2010 | Prospective Cohort | 11,092 | Diabetes, coronary heart disease, stroke and mortality | HbA1c had a stronger association with cardiovascular disease risk and mortality than fasting glucose in nondiabetic adults | Reliance on a single measurement of HbA1c and blood glucose at baseline and no long-term follow-up |
| Tong et al., 2022 | Retrospective analysis | 48,444 | The association between fasting plasma glucose and Gestational Diabetes Mellitus | Risk of GDM increased with FPG as low as 4.19 to 4.63 mmol/L (OR 1.137, p> 0.05) | FPG was only tested once per patient. Nutritional status data were not available. |

Supplementary Table 1b (ST 1b): Moderate-quality Evidence

| **Study** | **Design** | **Sample Size** | **Key Outcome** | **Main Finding** | **Limitations** |
| --- | --- | --- | --- | --- | --- |
| Cayonu et al., 2025 | Prospective Cohort | 404 | Diagnosis of Gestational Diabetes Mellitus and Adverse Obstetric Outcomes | Using an HbA1c cutoff of ≥ 5.7% was less accurate in diagnosing GDM than OGTT. HbA1c was also found to be associated with higher BMI and polyhydramnios | The results were not compared with those of patients who do not have a GDM diagnosis. |
| Ellis 2013 | Prospective Cohort | 50 | The accuracy of point-of-care testing for blood glucose | Capillary point-of-care testing showed unacceptable (88.3%) agreement with laboratory/arterial testing. The use of more than 2 medications significantly reduced the accuracy of arterial point-of-care testing. | Lack of multiple repeated measures |
| Ghazenfari et al., 2010 | Cross-sectional study | 604 | Association between HbA1c and FBS for diagnosis of T2DM | Analysis showed a strong and positive correlation between FBS and HbA1c | The study did not control for the impact of anaemia or abnormal haemoglobin on the HbA1c results. The study did not use multiple tests. |
| Juarez et al., 2014 | Systematic Review | 47 | Evaluating the strengths and weaknesses of HbA1c | HbA1c directly predicts microvascular complications and both convenient and effective in community and acute care settings | HbA1c may underestimate impaired glucose regulation in some ethnic groups and may not be valid for certain subgroups (children, pregnant women and patients with HIV or anaemia) |
| Martens et al., 2025 | Retrospective Cohort | 68 | CGM-based versus FBG-based basal insulin titration | CGM and FBG were equivalent for titration | The study does not consider hypoglycemia for down-titration and precludes comparison of hypoglycemic events |
| Rhee et al., 2019 | Retrospective Cohort | 942,446 | Prediction of T2DM | Random plasma glucose was significantly more accurate than demographic factors alone for predicting T2DM diagnosis within 3 to 5 years | The study was restricted to veterans, which has implications for RPG variability due to nutrition and stress |
| Vajravelu et al., 2023 | Longitudinal study | 176 | Performance of a non-fasting biomarker of dysglycemia | 1-h glucose challenge test was the strongest biomarker of dysglycemia in children and adolescents, while HbA1c and random glucose performed similarly | OGTT reproducibility was poor, and T2DM incidence was low. There was variation in follow-up time, which may have impacted the results |
| Xiao et al., 2025 | Systematic review and meta-analysis | 1,669 | HbA1c, fasting blood sugar, 2-h postprandial blood sugar and BMI | Improvements in blood glucose and BMI in patients with T2DM through digital diabetes management technology relative to tradictional care | Potential publuication bias due to heavy reliance on studies in China |
| Yao et al., 2020 | Retrospective Cohort | 568 | Predictive value of fasting plasma glucose on functional outcome in patients with acute ischemic stroke and diabetes | Fasting plasma glucose at admission was an independent predictor of poor functional outcomes and mortality | The study was conducted in a single facility. |
